# Pancreatic tuberculosis mimicking pancreatic cancer in immunocompetent patients: Case series and diagnostic pathways

**DOI:** 10.1016/j.radcr.2025.03.015

**Published:** 2025-03-31

**Authors:** Fakhrddine Amri, Kaoutar Chahi, Amal Mojahid, Abdelkrim Zazour, Hajar Koulali, Ouiam EL Mqaddem, Imane Skiker, Zahi Ismaili, Ghizlane Kharrasse

**Affiliations:** a Department of Hepato-Gastroenterology, Mohammed VI University Hospital, Oujda, Morocco; b Department of Radiology, Mohammed VI University Hospital, Oujda, Morocco; c Digestive Diseases Research Laboratory (DSRL), Faculty of Medicine and Pharmacy, Mohammed First University, Oujda, Morocco

**Keywords:** Pancreas, Mycobacterium tuberculosis, Diagnosis, Imaging, Endoscopic ultrasound, Fine-needle aspiration, Antituberculosis treatment, Case series

## Abstract

We retrospectively reviewed the clinical presentations, diagnostic evaluations, imaging findings, and treatment outcomes of 3 patients diagnosed with pancreatic tuberculosis. All 3 patients presented with nonspecific symptoms such as epigastric pain and weight loss. Imaging showed pancreatic masses suggestive of pancreatic cancer. EUS with FNA and subsequent histopathology confirmed tuberculosis through the presence of granulomas with caseous necrosis. All patients received anti-tuberculosis therapy, leading to favorable outcomes, including pain resolution and normalization of follow-up imaging.

## Introduction

Infection with Mycobacterium tuberculosis (MTB) remains one of the leading causes of mortality worldwide and continues to represent a significant public health issue, particularly in low- and middle-income countries [1]. Currently, tuberculosis is the 13th leading cause of death worldwide and the second leading cause of death from infectious diseases, after COVID-19. Patients infected with the human immunodeficiency virus (HIV) have an 18-fold increased risk of developing active tuberculosis. Furthermore, alcohol consumption and smoking increase the risk of tuberculosis by 3.3 and 1.6 times, respectively [2].

Abdominal tuberculosis accounts for 11%-16% of MTB cases, with the ileocecal region often being more affected than solid organs such as the kidney, spleen, and liver, and, in rare cases, the pancreas [3]. Pancreatic tuberculosis is extremely uncommon, even in areas with high tuberculosis prevalence, with reported incidences of less than 4.7% (14/297 cases) and 2% (11/526 cases) in other studies [4].

Diagnosing pancreatic tuberculosis is difficult due to its low incidence and nonspecific symptoms, which frequently resemble those of pancreatic cancer. Timely and accurate diagnosis is essential for initiating appropriate treatment. This not only facilitates effective pharmacological interventions in most cases but also helps prevent unnecessary and costly surgical procedures [5]. Herein, we present a case series of 3 patients diagnosed with pancreatic TB in our Gastroenterology department.

### Case 1

A 44-year-old woman, with no significant past medical history or known exposure to MTB, presented with complaints of atypical epigastric pain lasting for 2 months. The patient had previously undergone cholecystectomy for cholelithiasis several years prior, but there was no history of jaundice, fever, or unexplained weight loss. Her family history was noncontributory, with no known hereditary conditions or history of tuberculosis.

On clinical examination, there were no palpable abdominal masses, and no signs of peripheral lymphadenopathy. The patient's vital signs were stable, and the physical examination revealed no remarkable findings.

Routine laboratory tests were conducted, which revealed no significant abnormalities. The complete blood count (CBC) showed a white blood cell count (WBC) of 5900/mm^3^, with a lymphocyte count of 1300/mm^3^. Hemoglobin (Hb) was 12 g/dL, and the serum creatinine level was within the normal range at 6 mg/L. Liver function tests, including aspartate aminotransferase (AST) and alanine aminotransferase (ALT), were normal. Additionally, the total bilirubin and gamma-glutamyltransferase (GGT) levels were within normal limits. C-reactive protein (CRP) was normal at 2 mg/L, and the albumin level was 42 g/L. Carbohydrate antigen 19-9 (CA 19-9) was also normal (<2 IU/mL), as was the angiotensin-converting enzyme (ACE) level at 1 ng/mL. Sputum smears for acid-fast bacilli (AFB) were negative; however, the QuantiFERON test returned positive, raising the suspicion of tuberculosis as a potential underlying cause (Table 1).

**Table 1:** Summary of the demographic and baseline characteristics of the patients.

	Case 1	Case 2	Case 3	Normal range
Age	44	32	49	
Sexe	F	F	F	
Lipasemia	8	39	40	8-78 UI/L
Bilirubin	6	4	4	2-12 mg/L
AST	13	19	69	5-34 UI/L
ALT	16	10	30	0-55 UI/L
CRP	8	19	240	0-5 mg/L
WBC	5900	10,600	4600	4000-10,000/mm^3^
Lymphocyte	1300	2500	3900	1000-4500/mm^3^
HB	12	12	7.7	M 13-18 g/dL
				F 12-16 g/dl
Platelet count	223,000	333,000	237,000	150,000-400,000/µL
Creatinine	6	7	6	7-11 mg/L
Urea	0.19	0.25	0.26	0.15-0.45 g/L
Albumin	40	40	20	35-50 g/L
CA 19-9	<2	<2	6	
HIV	Negative	Negative	Negative	
MPD dilation	No	No	No	
Diagnosis	Surgery	EUS + FNA	Sputum (GeneXpert)	

**Fig. 1:**
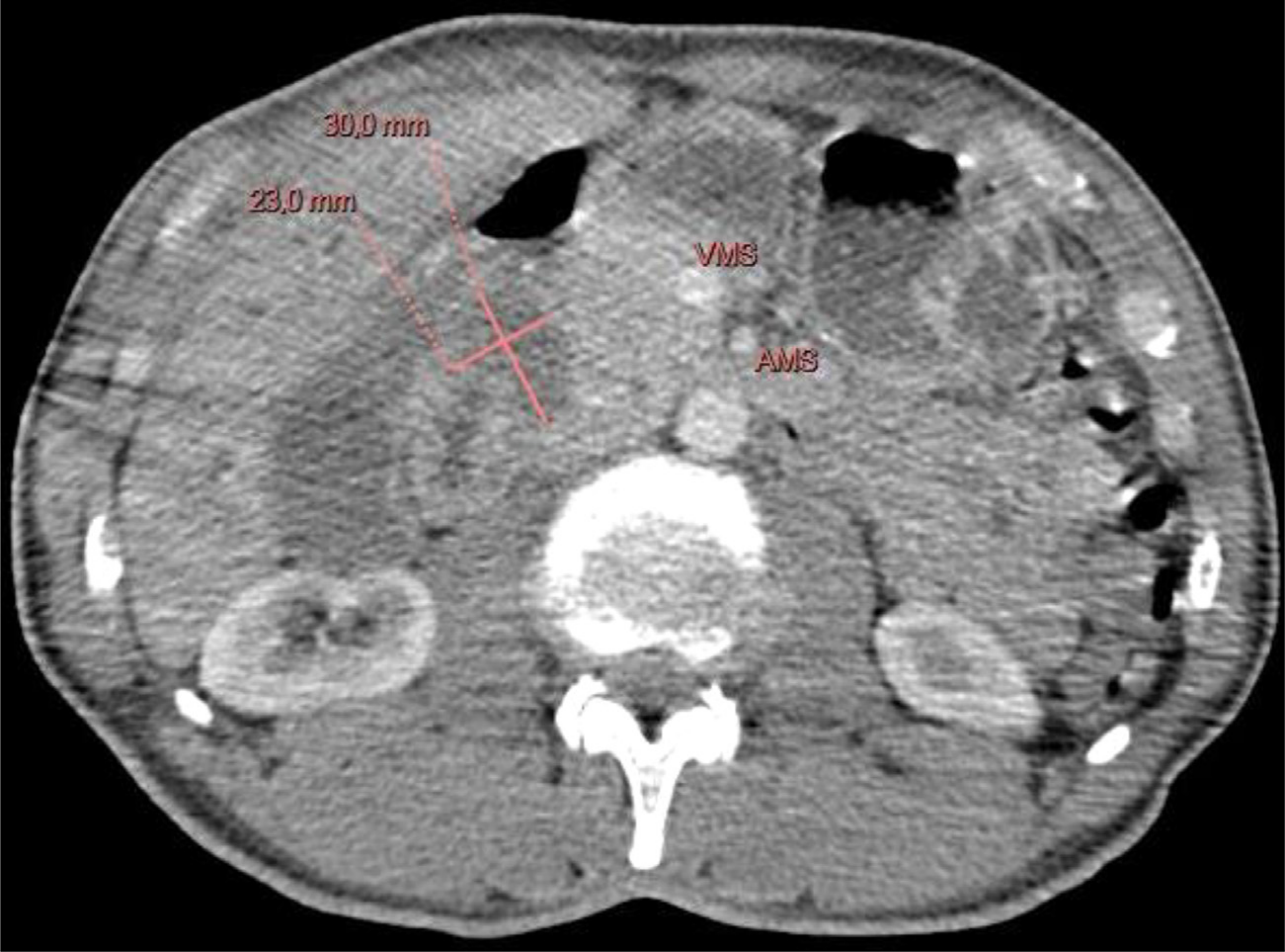
CT scan of the first case showing a pancreatic mass in the pancreatic head with no vascular invasion.

A series of imaging studies were performed to further evaluate the patient's condition. A chest, abdominal, and pelvic CT scan revealed a well-defined, heterogeneous mass in the head of the pancreas, measuring 23 × 30 mm. There was no evidence of vascular involvement, no ascites, and no pulmonary lesions (Fig. 1). Subsequently, an abdominal MRI was performed to gather further information. The MRI revealed a mass in the head of the pancreas measuring 46 × 39 mm, well-circumscribed, with specific characteristics on different imaging sequences:

• T1 sequence: The lesion appeared hypo-intense, with mild internal heterogeneity, suggesting a solid component and a central area of higher density. There was no marked contrast enhancement at this level.
• T2 sequence: The lesion appeared hyper-intense, with areas of higher signal intensity that may indicate necrosis or granulomatous inflammation.

Additionally, the MRI showed no dilation of the bile ducts or the main pancreatic duct (MPD), and no distant metastases were identified. The heterogeneous characteristics of the lesion were more apparent in the T2 sequence.

Endoscopic ultrasound (EUS) showed a 20 mm heterogeneous mass in the head of the pancreas, without dilation of the MPD. The mass was adjacent to the portal vein (PV), and there was a 30 mm lymph node at the hepatic hilum.

The imaging studies indicated a significant pancreatic mass that raised a strong suspicion of resectable pancreatic cancer. This urgency led to the decision to proceed with surgery without performing a cytological aspiration. The patient underwent an extended cephalic duodenopancreatectomy involving the body of the pancreas, along with retroportal and celiac trunk lymph node dissection. Histopathology showed chronic granulomatous pancreatitis, marked by epithelioid and giant cells. Granulomatous lymphadenitis was also detected, with similar findings of epithelioid and giant cells and punctate eosinophilic necrosis.

The patient was initiated on anti-tuberculosis treatment consisting of a 2-month regimen including rifampicin, isoniazid, pyrazinamide, and ethambutol, followed by 4 months of rifampicin and isoniazid alone (2RHZE/4RH).

The treatment resulted in favorable outcomes, with significant improvement in the patient's symptoms and overall health. Regular follow-up imaging showed no recurrence of the mass or any new lesions, indicating successful resolution of the disease. The patient reported relief from her initial epigastric pain and maintained stable vital signs, with no complications arising from the surgical intervention or the anti-tuberculosis therapy.

### Case 2

A 32-year-old female patient, without any notable medical history, she was previously healthy, with no known chronic conditions, and had no history of gastrointestinal disorders, pancreatitis, or any previous surgeries. The patient had no prior history of smoking, alcohol use, or substance abuse, and she was not on any regular medications. Regarding her family history, there was no known history of pancreatic diseases, tuberculosis, or any hereditary gastrointestinal disorders. She presented with atypical epigastric pain associated with weight loss. Clinical examination was unremarkable. Laboratory tests, including liver function tests, and tumor markers, were all within normal limits, however, WBC were elevated (10,600/mm^3^) as well as a CRP level of 19 mg/l, she had a slightly higher lipase level (39 UI/L), which did not suggest acute pancreatitis. However, The patient's liver enzymes, including AST and ALT, GGT and total bilirubine were within normal limits (Table 1).

**Fig. 2:**
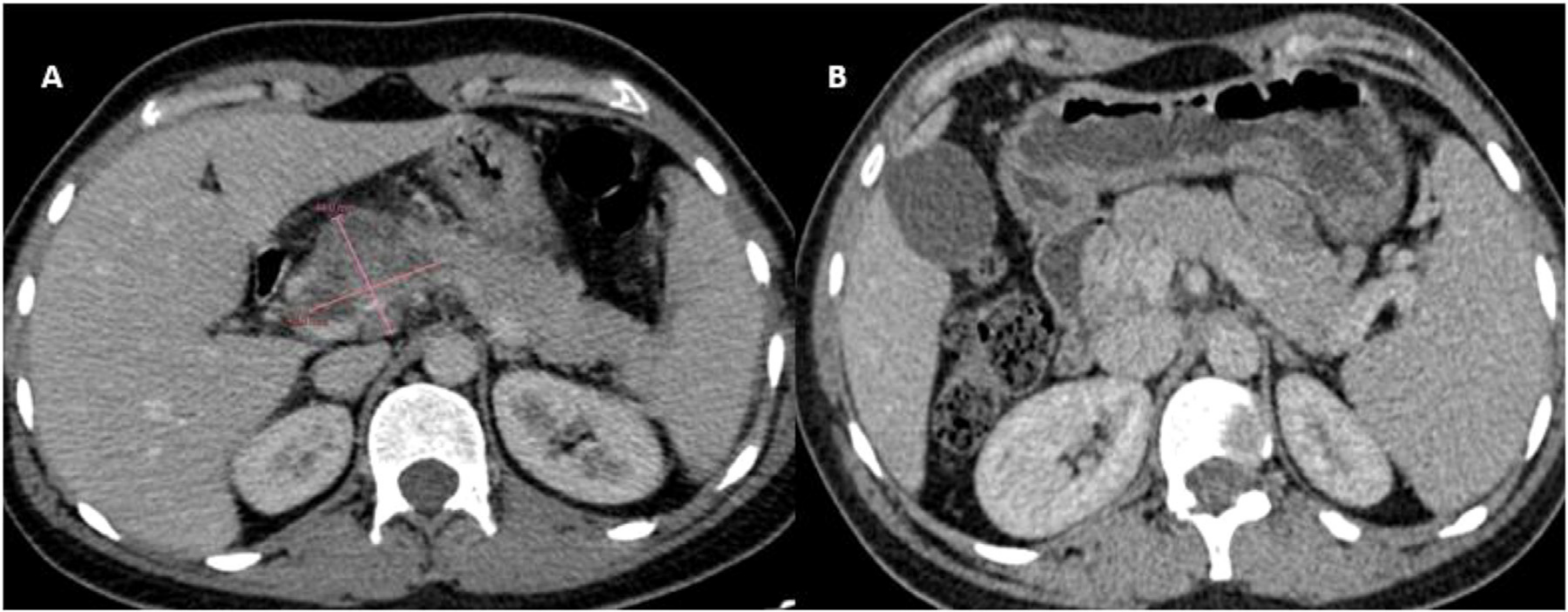
CT scan of the second case, transverse section showing (A) the pancreatic mass before anti-tuberculosis treatment, and (B) the pancreas after treatment.

The scan revealed a 50 mm mass located in the head of the pancreas. The mass exhibited invasion into the portal trunk, celiac trunk, and common hepatic artery, which raised concern for a locally advanced pancreatic process. The tumor was heterogeneous in appearance, with both solid and necrotic components. In addition to the mass itself, the CT scan also revealed coeliac lymphadenopathy, indicative of possible lymphatic spread or reactive lymph node enlargement due to the inflammatory process. However, no signs of distant metastasis were observed, as the lungs and liver. There were no signs of peripancreatic fluid collection or ascites, which could be seen in advanced malignancy or pancreatic abscesses (Fig. 2).

**Fig. 3:**
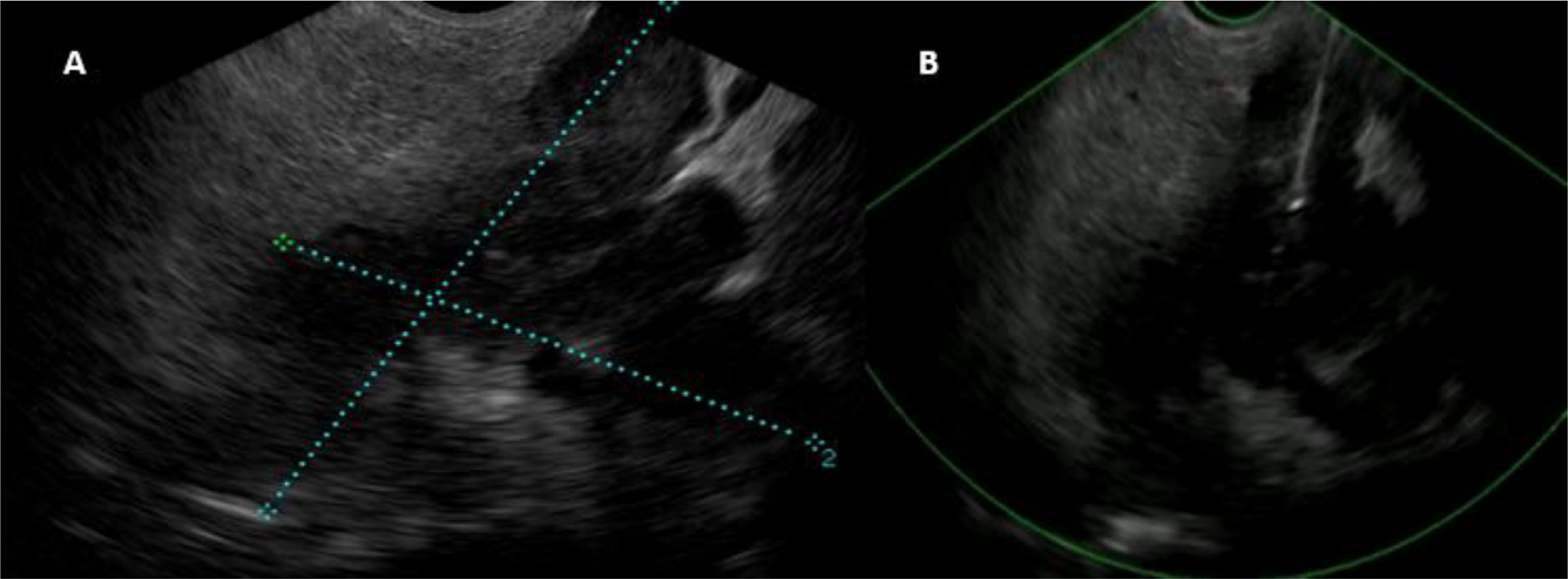
EUS of the second mass showing: (A) heterogeneous mass in the pancreatic head with irregular contours, (B): FNA.

**Fig. 4:**
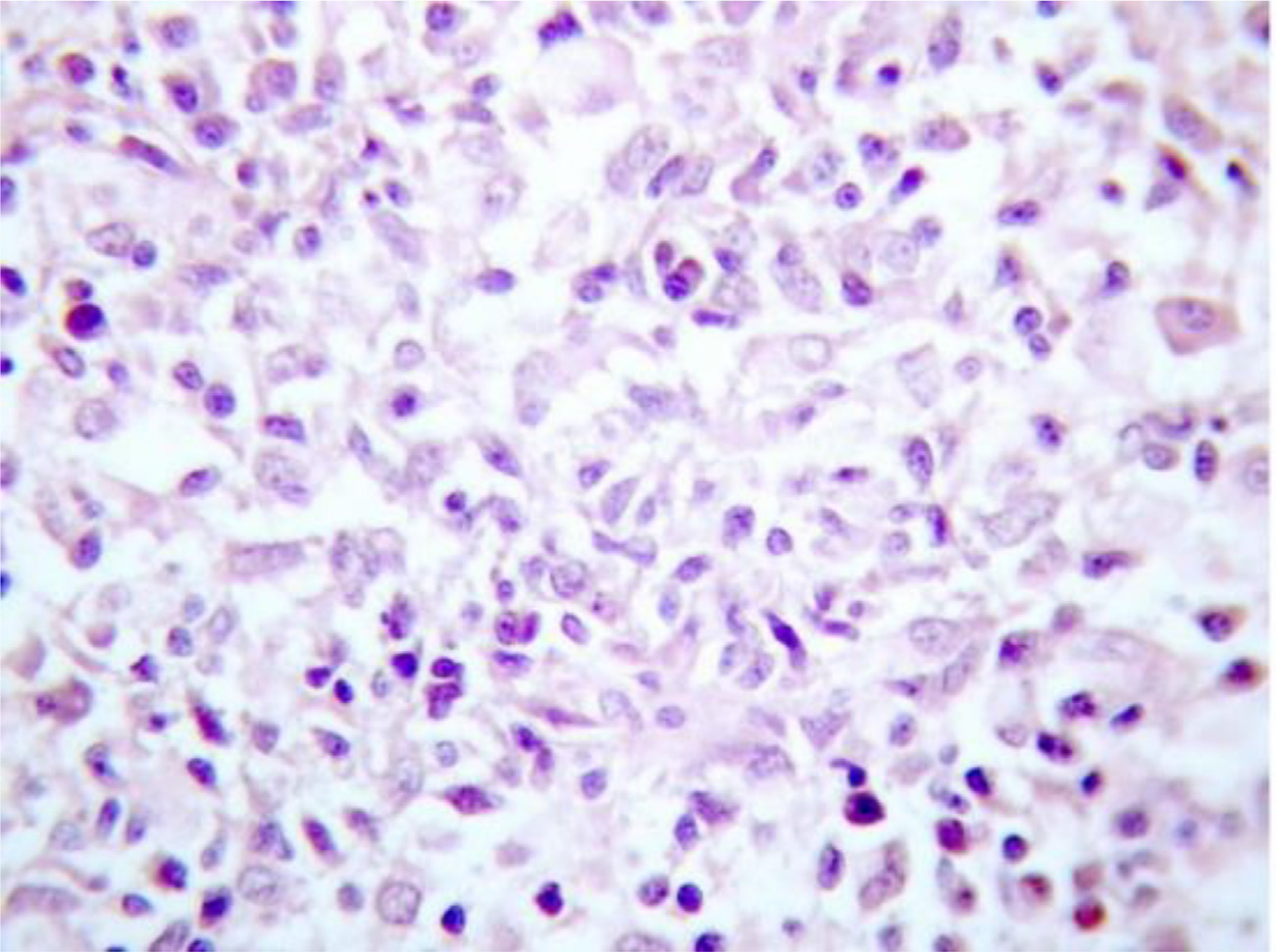
Photomicrograph of a cell block showing many aggregates of histiocytes in a necrotic and inflammatory background composed of lymphocytes and neutrophils, suggestive of a granulomatous inflammation (H&E, x400).

During EUS, a heterogeneous mass measuring 6 cm with irregular and nodular contours, along with necrotic areas, was noted in the head of the pancreas, appearing to invade the portal trunk. No dilation of the bile duct or MPD was observed (Fig. 3). Fine needle aspiration was performed. The histological appearance revealed extensive suppurative necrosis associated with a granulomatous reaction (Fig. 4). And the PCR analysis of the pancreatic fluid revealed the presence of Mycobacterium tuberculosis complex DNA, confirming the diagnosis of TB.

Following the diagnosis, the patient was started on an appropriate anti-tuberculosis treatment regimen. The standard treatment consisted of a 9-month course of anti-tuberculosis medications, including rifampicin, isoniazid, pyrazinamide, and ethambutol during the initial 2 months, followed by rifampicin and isoniazid for the remaining 7 months. Throughout the treatment period, the patient was monitored regularly for clinical improvement and for potential side effects of the anti-tuberculosis medications. She experienced a gradual resolution of her symptoms, with epigastric pain significantly improving and weight gain observed over time. Regular blood tests showed a steady decline in her CRP levels. At the 9-month follow-up, a follow-up CT scan was performed and revealed a complete disappearance of the previously seen mass in the head of the pancreas, with no evidence of any structural abnormalities, and the coeliac lymphadenopathy that had been observed on the initial CT scan also resolved.

### Case 3

A 49-year-old female presented to our department with atypical epigastric pain associated with vomiting that had persisted for one month. She also reported a productive cough, fever, and unintentional weight loss over the same period. These symptoms raised concern for a possible systemic infection. The patient had a past medical history of treated ophthalmic herpes zoster, and importantly, she had a family history of pulmonary tuberculosis (TB), with her mother having been diagnosed with and treated for pulmonary TB years ago.

On clinical examination, the patient's vital signs were normal (e.g., heart rate, blood pressure, temperature). Epigastric tenderness was noted on palpation, but there was no palpable lymphadenopathy. Additionally, bilateral pleural effusion was detected upon auscultation. There was no significant abdominal distension or signs of acute abdomen, which helped to rule out other urgent gastrointestinal conditions. the laboratory results revealed significant findings indicative of a systemic inflammatory process. The Hb was low at 7.7 g/dL, suggesting anemia. CRP was markedly elevated at 240 mg/L, indicating systemic inflammation. The patient also had hypoalbuminemia with an albumin level of 20 g/L.

Other values were within normal limits: the WBC count was 4600/mm³, the lymphocyte count was 3900/mm³, and the platelet count was 237,000/µL. Renal function and liver enzymes, including creatinine, lipase, bilirubin, AST, and ALT, were all normal. The CA 19-9 level was 6 IU/mL, slightly elevated, but nonspecific. The HIV test was negative

**Fig. 5:**
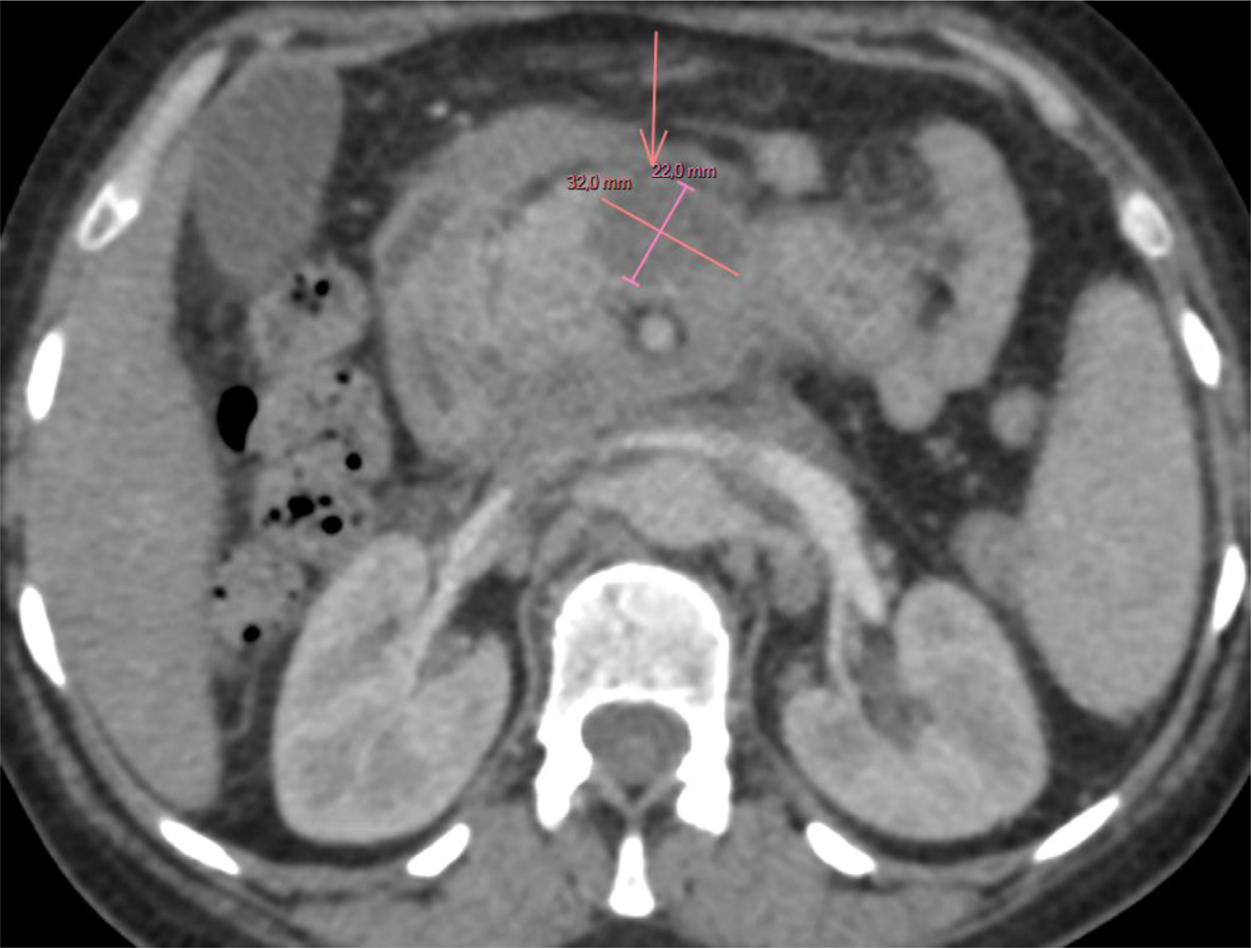
CT scan of the third case, transverse section, demonstrating hypertrophy of the pancreatic gland with a cystic area.

The CT scan showed pancreatic hypertrophy with a cystic necrotic area, moderate ascites with retroperitoneal lymph nodes (Fig. 5), hepatomegaly, bilateral pleural effusion, and bilateral pulmonary parenchymal micronodules.

To confirm the suspected diagnosis of tuberculosis, pleural fluid analysis was performed due to the detected pleural effusion. The fluid was exudative, with a neutrophilic predominance, suggesting an inflammatory process. Further investigation with GeneXpert testing on sputum samples returned positive for MT) DNA, confirming the diagnosis of miliary tuberculosis.

Once the diagnosis of miliary tuberculosis was established, the patient was immediately started on a standard anti-tuberculosis regimen. This regimen included the four-drug combination of rifampicin, isoniazid, pyrazinamide, and ethambutol for the initial 2 months, followed by rifampicin and isoniazid for the remaining 7 months, according to the standard treatment protocol for miliary tuberculosis.

The patient was monitored closely for clinical and laboratory improvements. Over time, she reported significant relief from her symptoms, including the epigastric pain and vomiting. She also experienced improvement in her general condition, with weight gain and reduction in systemic inflammatory markers. The pleural effusion began to resolve, and follow-up imaging confirmed a decrease in the pancreatic cystic necrosis, lymphadenopathy, and pulmonary micronodules.

## Discussion

Tuberculosis continues to be a major infectious disease globally, the rise of HIV/AIDS has led to a resurgence of tuberculosis as an opportunistic infection, particularly in developed nations [4]. The most prevalent form of extrapulmonary tuberculosis is lymphoid involvement. abdominal tuberculosis ranks as the sixth most common type [6]. The biliary tree or neighboring pancreatic tissue are less affected, largely due to the antimicrobial properties of pancreatic enzymes like lipase [7]. The uniqueness of our case lies in the occurrence of pancreatic TB in immunocompetent patients.

The pathophysiology of pancreatic tuberculosis typically involves hematogenous or lymphatic spread, or direct extension from neighboring organs. Three distinct forms have been identified: (1) as part of miliary tuberculosis, the most common form; (2) through spread from retroperitoneal lymph nodes to the pancreas; or (3) as an isolated pancreatic manifestation [4].

According to various published studies, pancreatic tuberculosis primarily affects men, with a higher incidence observed during the fourth and fifth decades of life [5]. This could be explained by the higher prevalence of risk factors in this demographic, particularly smoking, alcohol consumption, and HIV infection. However, in our series, all cases are female.

Diagnosis is often delayed due to the nonspecific nature of clinical symptoms. A meta-analysis of 116 studies with 166 patients indicated that abdominal pain was present in 74.8% of cases, fever in 46.5%, weight loss in 51.6%, jaundice in 20%, and diarrhea in 3.1% [3].

Anemia, liver function abnormalities, and changes in serum bilirubin or glucose levels may be detected in laboratory tests. The diagnostic utility of serum amylase and lipase levels is limited, as these markers are elevated in only a fraction of pancreatic tuberculosis cases (26.8% and 31.3%, respectively) [8].

The radiological diagnosis in our series was challenging, particularly in the first 2 cases, as they exhibited features suggestive of pancreatic cancer, leading to a pancreaticoduodenectomy in the initial case. The same challenge was reported in the literature, due to nonspecific radiological findings [7]. CT scans were used in nearly all reported cases of pancreatic tuberculosis in the literature, as pancreatic masses were observed in 79.5% of cases, most commonly located in the pancreatic head (59%) [9]. Some diagnostic clues may stand out. Even though the mass is centrally situated in the head of the pancreas, the common bile duct and MPD often show no abnormalities [10]. The absence of vascular involvement may be a suggesting indicator of pancreatic tuberculosis, however, it cannot reliably serve as a distinguishing factor, as multiple reports indicate vascular invasion. Others findings suggestive of tuberculosis are: the absence of distant metastasis and the presence of necrotic areas in the mass [11,12] (Table 2).

**Table 2:** Comparison of imaging findings in pancreatic tuberculosis and pancreatic cancer.

Imaging modality	Pancreatic tuberculosis	Pancreatic cancer
CT findings	Well-defined mass; no vascular invasion; possible lymphadenopathy	Irregular mass; vascular invasion; possible metastases
MRI findings	Hypo-intense on T1; hyper-intense on T2; granulomatous features	Hypo-intense mass with irregular contours; marked enhancement
EUS findings	Heterogeneous mass; no MPD dilation; possible lymphadenopathy	Irregular, nodular mass; possible MPD obstruction

Endoscopic ultrasound with aspiration (FNA)or biopsy (FNB) has emerged as the preferred minimally invasive technique for diagnosing pancreatic tuberculosis, thus helping to avoid unnecessary surgery [13]. Firstly, it can differentiate between pancreatic and peripancreatic masses, as well as identify abdominal and mediastinal lymphadenopathy. EUS has a diagnostic yield of up to 95% for pancreatic cancer and up to 76% for pancreatic TB [14]. FNA (B) can be performed on pancreatic masses or lymphadenopathy, with the samples undergoing histological and microbiological analysis, including Ziehl–Neelsen staining, acid-fast bacilli culture, and polymerase chain reaction (PCR) testing [15]. Histopathological examination for diagnosis typically involves identifying granulomas with caseation necrosis and multinucleated giant cells, or the detection of acid-fast bacilli [7].

ERCP (Endoscopic Retrograde Cholangiopancreatography) is not routinely recommended for the diagnosis or management of pancreatic TB. However, it may be performed in certain situations, such as to alleviate biliary obstruction in patients, particularly when ductal narrowing persists despite anti-tuberculosis treatment [18].

Treatment for pancreatic tuberculosis follows the same protocol as for other forms of extrapulmonary tuberculosis [16], it's include a 4-fold combination: isoniazid (5 mg/kg BW/day), rifampicin (10 mg/kg BW/day), pyrazinamide (30 mg/kg BW/day), and ethambutol (20 mg/kg BW/day), generally administered for 2–4 months. Following this, a continuation phase with isoniazid and rifampicin for 6-12 months is recommended [16]. For follow-up and to guide clinicians on the complete resolution and duration of therapy, CT imaging serves as a valuable tool [17].

The prognosis for pancreatic TB is generally favorable with appropriate treatment. Patients typically respond well to standard anti-tuberculosis regimens. Regular follow-up imaging, such as CT scans, is essential to evaluate treatment response, with positive indicators including a decrease in mass size, symptom resolution, and normalization of laboratory values. Long-term surveillance in our case series demonstrated no recurrence or residual pancreatic abnormalities, suggesting that successful treatment leads to significant improvement in quality of life and low recurrence rates. However, potential complications such as abscess formation or biliary obstruction may arise, necessitating additional interventions. Demographic factors, including gender and age, can influence outcomes, with patients having comorbidities like diabetes or HIV requiring closer monitoring

## Conclusion

Pancreatic tuberculosis remains a challenging diagnosis, owing to its rarity, nonspecific symptoms, and imaging findings that can resemble those of pancreatic cancer. Nevertheless, a high index of suspicion is essential, particularly in endemic countries and in patients with known risk factors. The use of advanced diagnostic modalities, including EUS with FNA, is crucial for accurate diagnosis. Once identified, timely initiation of anti-tuberculosis treatment can result in favorable outcome.

## Ethics approval and consent to participate

Ethics committee approval was waived.

## Consent for publication

Written informed consent was obtained from the patients for publication of this case report and any accompanying images. A copy of the written consent is available for review by the Editor-in-Chief of this journal.

## Author contributions

Writing and analysis: Amri F, Correction: Kharrasse G, Data source: Ismaili Z, Skiker I, Zazour A, Koulali H, El Mqaddem O, Mojahid A, Chahi Kawtar.

## Patient consent

Informed consent was obtained from all patients for publication.
